# Examining the Mental Health, Wellbeing, Work Participation and Engagement of Medical Laboratory Professionals in Ontario, Canada: An Exploratory Study

**DOI:** 10.3389/fpubh.2022.876883

**Published:** 2022-07-15

**Authors:** Behdin Nowrouzi-Kia, Jingwen Dong, Basem Gohar, Michelle Hoad

**Affiliations:** ^1^Department of Occupational Science and Occupational Therapy, Temerty Faculty of Medicine, University of Toronto, Toronto, ON, Canada; ^2^Krembil Research Institute, University Health Network, Toronto, ON, Canada; ^3^Centre for Research in Occupational Safety and Health, Laurentian University, Sudbury, ON, Canada; ^4^School of Public Health, Shanghai Jiao Tong University, Shanghai, China; ^5^Department of Population Medicine, The University of Guelph, Guelph, ON, Canada; ^6^Medical Laboratory Professionals Association of Ontario, Hamilton, ON, Canada

**Keywords:** medical laboratory technologists, medical laboratory assistants/technicians, medical laboratory professionals, mental health, occupational health, Canada

## Abstract

**Objectives:**

The overall objective of this proposed project is to examine the impact of the COVID-19 pandemic on the mental health, functioning and wellbeing of medical laboratory technologists (MLT) and medical laboratory technicians/assistants (MLT/A) in Ontario, Canada.

**Methods:**

A cross-sectional study included a self-reported questionnaire for MLT and MLT/A in Ontario. The questionnaire included questions about demographics and occupational characteristics. Questions about mental health, functioning, well-being and psychosocial work environments were also included using validated questionnaires.

**Results:**

There were 551 MLT and 401 MLT/A in the analytic sample. Most of the respondents were women. The mean age and standard deviation of the overall sample were 42.0 ± 11.8. MLT demonstrated higher quantitative demands, possibilities for development, and organizational justice compared to MLT/A. The scores of work pace, emotional demands, role conflicts, job insecurity, insecurity over working conditions and negative acts were higher for MLT/A than MLT. The WHODAS 2.0 scores of the respondents were 20.80 ± 6.68, higher than approximately 92% average people. For both groups, most respondents scored the COPSOQ-III domains as worse since COVID-19.

**Conclusion:**

The study provides preliminary evidence regarding the workplace mental health outcomes of medical laboratory professionals in Ontario, Canada. The findings suggest that MLT and MLT/A experience psychosocial work conditions that impact mental health, functioning and disability. Accordingly, additional research is necessary to understand the experiences of medical laboratory professionals.

## Introduction

In Canada, Medical Laboratory Technologists (MLT) and Medical Laboratory Technicians/Assistants (MLT/A) are the sixth-largest groups of healthcare professionals. MLT job duties include laboratory analyses, where physicians use the results to evaluate and make informed decisions about their patients' health and possible treatment (1). Working with MLT, MLT/A work responsibilities include conducting medical laboratory testing and maintaining equipment. During the COVID-19 pandemic, MLT and MLT/A have been responsible for the processing of COVID-19 tests (2). In Ontario, MLT and MLT/As are experiencing increased workloads because of the COVID-19 pandemic, leading to deleterious health outcomes, including burnout (3–6). In addition, Ontario is facing a severe shortage of MLT in 2019, the Medical Laboratory Professionals' Association of Ontario (MLPAO) indicated a significant labor shortage of MLT in Ontario (7). According to the MLPAO (2), 70% of labs that entered COVID-19 were already short-staffed. Furthermore, 44% of MLT will retire in the next 4–8 years (7). The situation is worse in rural and remote areas, where nearly half (45%) of employment opportunities are located. The shortage is ongoing as 39% of openings have been on the market for more than 12 months. Moreover, the healthcare system cannot meet this labor shortage. The increased workload exacerbates this situation and leads to increased work exhaustion, job dissatisfaction, and turnover rates. This leads to mental health illness and stress creating a constant cycle of increased workload, poor mental health, staff illness and absences, and staff shortages (8).

Poor psychosocial health safety has been associated with adverse health outcomes among workers (6). Key workplace factors that can influence psychological wellbeing include collegial relationships, workplace supports and resources, autonomy, working conditions, and opportunities for and attainment of achievements. An example of a health outcome is job stress. In a study with 4,613 laboratory professionals, 96.1% reported feeling “a little bit to a lot of stress” (9). Respondents also reported feeling overwhelmed by their workload, having little control over their work schedule, and feeling anxious about work. Another study found that the likelihood of high job stress for MLT is 36%, compared to nurses and nurse supervisors (67%) and MLT/A (64%) (10). Occupational burnout is another negative workplace health outcome, which is caused by prolonged job stress and leads to physical and emotional exhaustion. Factors associated with burnout and job stress are understaffing and high workloads. Burnout has been studied extensively in many health care groups, including doctors (5, 11, 12), nurses (6, 9, 12), occupational and physical therapists (8, 13–15), psychologists and social workers (4). However, there is a dearth of evidence examining burnout among MLT and MLT/A, particularly in Canada. Other areas of psychosocial wellbeing include support resources, job satisfaction, and emotional demands. Moreover, these factors can impact functioning, biopsychosocial health, job satisfaction, productivity, and work performance (9).

Several studies discuss the significant impact of COVID-19 pandemic on the mental health of individuals, causing stress, anxiety, depressive symptoms, insomnia, denial, anger, and fear (16, 17). More specifically, the mental health of frontline health workers during the COVID-19 pandemic have been widely reported and include symptoms of psychological distress, burnout, anxiety and stress (18–21). The pandemic has increased workplace demands and stressors to healthcare professionals as they had to adapt to increased workloads, new work practices including telehealth and personal protective equipment, increased risks to infection, and redeployment (18). In a national U.S. survey looking at cardiac catheterization laboratory (CCL) nurses and technologists during the COVID-19 pandemic, there was an increase in anxiety/stress (80%), fear (39%), depression (36%), and anger (38%) (22). Furthermore, in a study with healthcare professionals, they found that the increasing number of cases, shortage of medical staff, build-up of working hours and the latent risk of infection were among many factors that increased internal stress (23). In a survey of 348 Ontario laboratory professionals, 87% reported experiencing burnout after a year of testing 24/7 (24).

The literature suggests that prolonged exposure to deleterious mental health and functional outcomes is associated with negative physical and mental health outcomes (24–28).

The literature also suggests that the COVID-19 pandemic has negative effects on mental health (17). The impact of the pandemic on MLT and MLT/A mental health and functioning includes but is not limited to their psychosocial work environment (e.g., work stress, job satisfaction, work productivity, quality of work-life, and work demands) and their means of coping, which remains poorly understood. Thus, the overall objective of this proposed project is to examine the impact of the COVID-19 pandemic on the mental health, functioning and wellbeing of medical laboratory technologists (MLT) and medical laboratory technicians/assistants (MLT/A) in Ontario.

## Methods

### Design

A cross-sectional study included a self-reported questionnaire for MLT and MLT/A in Ontario, Canada. The questionnaire was developed in close collaboration with the MLPAO, medical laboratory professionals and a comprehensive literature review. The questionnaire included questions about MLT and MLT/A mental health, wellbeing and psychosocial work environments, roles and demographics, and occupational characteristics using validated questionnaires. The research project was approved by the research ethics board at the University of Toronto. All the data will be collected and securely stored on REDCap (29) servers at the University of Toronto. The study received ethical approval from the University of Toronto Research Ethics Board (*ID censored for peer-review*).

### Sample

MLT and MLT/A were invited to complete an anonymous questionnaire and informed consent stating the study's objectives, description, respondents' rights as research participants. In addition, the respondents were provided with two electronic reminders 2 and 4 weeks after the launch of the questionnaire to those who had not responded to the survey. The MLPAO is a provincial organization that represents the interests of medical laboratory professionals with government, health care professionals, regulatory bodies and academic institutions (24). The questionnaire and all reminders were distributed electronically by the MLPAO.

All MLT and MLT/A who met the following eligibility criteria were invited to participate in the current study: (1) actively registered with the College of Medical Laboratory Technologists of Ontario (only for MLT who are a regulated health care profession in the province), (2) Ontario was their clinical practice location, (3) employed and working as of March 11, 2020 (start date of the global pandemic) and (4) position as an MLT or MLT/A providing direct or indirect clinical patient care. In total, there were 553 MLT and 401 MLT/A that met the study's eligibility criteria. We applied a sample size calculation (30) to determine the sample size that was adequate to detect small to moderate differences in the level of job stress and burnout as perceived for MLT and MLT/As working across Ontario, Canada.

### Measures

The questionnaire included items from the Copenhagen Psychosocial Questionnaire, third edition (COPSOQ-III) English version, an instrument designed for assessing psychosocial conditions in the workplace (31). The present study used 48 questions from the COPSOQ-III middle version, to measure mental health, participation and engagement in MLT and MLT/A. The COPSOQ-III looks at the following domains: work demands, work organization and job contents, interpersonal relations and leadership, work-individual interface, social capital, health and wellbeing and negative acts. Twenty-seven dimensions were assessed across these domains, including but not limited to social support, job security, burnout, and stress. For each COPSOQ-III dimension, Likert Scale–type items were measured and scaled to the interval of 0–100. Response options vary by scale values and scale direction. For example, *Very satisfied (100), Satisfied (75), Neither/Nor (50), Unsatisfied (25), Very unsatisfied (0)*. Psychometric properties of the COPSOQ-III were assessed in various countries, including Canada (31). In an international study looking at the COPSOQ-III psychometric properties, ceiling effects were present for the dimensions Sense of Community at work (30%), Social Support from Colleagues (21%), Social Support from Supervisor (25%), Meaning of Work (25%) and Quality of Work (26%). Furthermore, floor effects were present for dimensions such as Job Insecurity (19%). The COPSOQ-III also has Canadian standardized used to compare to our population. As this portion of the study is cross-sectional, it is unknown if the results are influenced by COVID-19. To this end, following each domain, the participant answered the question: “Since COVID-19, my current response is ‘better than, ‘the same as', or ‘worse than' before the pandemic.”

The World Health Organization Disability Assessment Schedule (WHODAS) 2.0 was used to measure functioning and disability in MLT and MLT/A. This 36-item questionnaire covers 6 domains of functioning: 1 = cognition, 2 = mobility, 3 = self-care, 4 = getting along, 5 = life activities, and 6 = participation (32). The WHODAS 2.0 uses a Likert-like scale and scores for each item 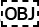
ranges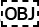 rom 1 (none) to 5 (extreme). Psychometric properties of the WHODAS 2.0 have been assessed in various countries, including Canada (33, 34). In addition, the factor structure is consistent across different health populations such as psychiatric illnesses, sclerosis, stroke, and alcohol dependency.

### Data Analysis

All statistical analyses were performed using R software (Version 4.1.0 for Windows) (35). Demographic information was summarized using descriptive statistics including percentages, means and standard deviations. The current status of mental health, functioning, and wellbeing, and the impact of the COVID-19 pandemic on MLT and MLT/A was examined using descriptive and inferential statistics. To examine the differences in COPSOQ domains, we dichotomized the scores into “low” and “high” by using the median values of the distribution (36). Analysis of variance (ANOVA) and *t-*test were used to examine differences between groups. Comparisons between some groups were made using Fisher's exact analysis due to the small number of certain groups. The sample size varied for some analyses because of missing data on covariates, and the numbers were indicated in the tables. Statistical significance was set at *p* < 0.05.

## Results

The study's response rate was 79.6% (954/1198). The majority (89.9%) of the respondents, including 88.9% MLT and 91.3% MLT/A, self-identified as women. The mean age and standard deviation of the overall sample, MLT and MLT/A were 42.0 ± 11.8, 43.9 ± 12.3, 39.5 ± 10.5, separately. High proportion (77.6%) of Caucasian/white was the characteristic for the considered study population (accounting for 84.8% MLT and 67.6% MLT/A). Table 1 summarizes the main demographic characteristics of the study population.

**Table 1:** Demographic and occupational characteristics of MLT and MLT/A respondents.

	**MLT (*n* = 553)**		**MLT/A (*n* = 401)**		**Overall (*n* = 954)**	
**Gender (*n* =953)**						
Male	59	10.7%	33	8.2%	92	9.7%
Female	491	88.9%	366	91.3%	857	89.9%
Other	2	0.4%	2	0.5%	4	0.4%
**Age group (*n* = 906)**						
18–35	175	33.6%	142	36.9%	317	35.0%
36–49	145	27.8%	163	42.3%	308	34.0%
50 and over	201	38.6%	80	20.8%	281	31.0%
**Marital status (*n* = 952)**						
Single	83	15.0%	95	23.8%	178	18.7%
Married/common law/ committed	428	77.4%	274	68.7%	702	73.7%
Separated/divorced /widowed	42	7.6%	30	7.5%	72	7.6%
**Highest level of education attained (*n* = 954)**						
High school	17	3.1%	67	16.7%	84	8.8%
Community college	239	43.2%	229	57.1%	468	49.1%
University	285	51.5%	99	24.7%	384	40.2%
Other	12	2.2%	6	1.5%	18	1.9%
**Ethnicity (*n* = 954)**						
Caucasian/white	469	84.8%	271	67.6%	740	77.6%
Other	84	15.2%	130	32.4%	214	22.4%
**Number of children living at home (*n* = 927)**						
0	296	54.5%	177	46.1%	473	51.0%
1	73	13.4%	69	18.0%	142	15.3%
2	141	26.0%	88	22.9%	229	24.7%
3	24	4.4%	37	9.6%	61	6.6%
4	9	1.7%	7	1.8%	16	1.8%
5	0	0.0%	6	1.6%	6	0.6%
**Accommodation required at work due to disability (*n* = 950)**						
Yes	23	4.2%	10	2.5%	33	3.5%
No	516	93.6%	367	92.0%	883	92.9%
Prefer not to answer	12	2.2%	22	5.5%	34	3.6%
**Employment status (*n* = 948)**						
Full-time	445	80.6%	255	64.4%	700	73.9%
Part-time	93	16.9%	113	28.5%	206	21.7%
Other	14	2.5%	28	7.1%	42	4.4%

### COPSOQ Scales

The mean values for the COPSOQ III scales and a comparison of psychosocial factors based on the domains and dimensions of COPSQQ-III among the respondents were presented in Table 2.

**Table 2:** A comparison of psychosocial factors of the respondents based on COPSOQ-III dimensions.

**COPSOQ-III domain** ***(# of dimensions)***	**Dimension**	**Medical laboratory technologists** **(*N* = 553)** **(Mean ±SD)**	**Medical laboratory technicians/assistants** **(*N* = 401)** **(Mean ±SD)**	**Total** **(Mean ±SD)**	**Comparison between groups**
Demands at Work (3)	Quantitative demands	59.8 ± 23.5	55.9 ± 25.5	58.4 ± 24.3	*p* < 0.05^*^
	Work pace	79.2 ± 17.9	83.1 ± 17.3	80.6 ± 17.8	*p* < 0.01^**^
	Emotional demands	61.5 ± 23.2	66.4 ± 22.4	63.2 ± 23.0	*p* < 0.01^**^
Work organization and job	Influence at work	34.5 ± 21.1	32.2 ± 23.9	33.7 ± 22.1	*p* = 0.20
contents (3)	Possibilities for development	66.0 ± 19.2	60.6 ± 20.5	64.0 ± 19.8	*p* < 0.001^***^
	Meaning of work	83.4 ± 18.2	81.1 ± 20.2	82.5 ± 19.0	*p* = 0.15
Interpersonal relations and	Predictability	46.0 ± 20.7	46.2 ± 24.3	46.1 ± 22.1	*p* = 0.91
leadership (8)	Recognition	47.6 ± 25.5	45.8 ± 26.7	47.0 ± 26.0	*p* = 0.39
	Role clarity	70.1 ± 19.3	71.3 ± 19.3	70.5 ± 19.3	*p* = 0.43
	Role conflicts	50.4 ± 25.0	55.3 ± 24.2	52.1 ± 24.8	*p* < 0.05^*^
	Quality of leadership	46.4 ± 27.1	48.0 ± 26.5	47.0 ± 26.9	*p* = 0.43
	Social support from supervisor	51.8 ± 27.2	52.7 ± 28.1	52.2 ± 27.5	*p* = 0.69
	Social support from colleagues	66.6 ± 22.7	64.4 ± 26.4	65.8 ± 24.1	*p* = 0.28
	Sense of community at work	71.1 ± 19.9	67.8 ± 22.9	69.9 ± 21.0	*p* = 0.06
Work–individual interface (4)	Job insecurity	20.0 ± 25.3	36.7 ± 31.9	26.0 ± 29.0	*p* < 0.001^***^
	Insecurity over working conditions	21.4 ± 29.7	27.2 ± 33.9	23.5 ± 31.4	*p* < 0.05^*^
	Job satisfaction	59.4 ± 28.3	59.9 ± 26.1	59.6 ± 27.5	*p* = 0.83
	Work life conflict	63.8 ± 27.9	65.2 ± 28.2	64.3 ± 28.0	*p* = 0.54
Social Capital (2)	Vertical trust	59.6 ± 21.4	62.4 ± 20.6	60.6 ± 21.2	*p* = 0.09
	Organizational justice	50.5 ± 21.3	46.2 ± 22.8	49.0 ± 21.9	*p* < 0.05^*^
Health and well-being (3)	Self-rated health	59.7 ± 22.9	60.2 ± 25.7	59.9 ± 23.9	*p* = 0.80
	Burnout	70.1 ± 23.6	71.4 ± 25.9	70.6 ± 24.5	*p* = 0.52
	Stress	64.0 ± 22.6	65.2 ± 24.4	64.4 ± 23.2	*p* = 0.52
Negative acts (4)	Sexual harassment	1.6 ± 7.0	4.0 ± 10.7	2.4 ± 8.6	*p* < 0.01^**^
	Threats of violence	2.8 ± 9.5	13.8 ± 22.7	6.7 ± 16.5	*P* < 0.001^***^
	Physical violence	1.0 ± 5.1	7.8 ± 17.8	3.4 ± 11.9	*p* < 0.001^***^
	Bullying	16.0 ± 24.4	19.7 ± 27.1	17.3 ± 25.4	*p* = 0.08

* *p < 0.05*,

** *p < 0.01,*

*** *p < 0.001*.

### Demands at Work

The results showed that the scales of “quantitative demands” were significantly higher for the group of MLT (59.8 ± 23.5) than MLT/A (55.9 ± 25.5). High work pace (79.2 ± 17.9) and emotional demands (61.5 ± 23.2) for the group of MLT were determined, but they were lower than those for MLT/A (83.1 ± 17.3 and 66.4 ± 22.4, respectively).

### Work Organization and Job Contents

The values of the “possibilities for development” dimension were higher for MLT (66.0 ± 19.2) than MLT/A (64.0 ± 19.8). Values of influence at work and meaning of work didn't have any distinction between the two groups.

### Interpersonal Relations and Leadership

Compared to MLT (50.4 ± 25.0), the values of role conflicts for MLT/A were significantly higher (55.3 ± 24.2). Besides role clarity and role conflicts, other six dimensions (predictability, recognition, quality of leadership, social support from supervisor, social support from colleagues, sense of community at work) presented relatively lower scores than general Canadian workers.

### Work-Individual Interface

The categories “job insecurity” and “insecurity over working conditions” for MLT/A were rated high (36.7 ± 31.9 and 27.2 ± 33.9), while MLT rated these two dimensions within the relatively normative range (20.0 ± 25.3 and 21.4 ± 29.7).

### Other Domains

MLT hold significantly higher scores (50.5 ± 21.3) in organizational justice than MLT/A (46.2 ± 22.8). MLT/A experienced more sexual harassment, threats of violence and physical violence than MLT.

### WHODAS 2.0 Scores

Table 3 demonstrates the scores of the WHODAS 2.0 among respondents across the six domains. We found that a mean and standard deviation score of 20.80 ± 6.68 on the WHODAS 2.0 was the best score for describing the MLT and MLT/A population, 20.38 ± 6.34 for MLT and 21.59 ± 7.22 for MLT/A, separately. The participant scores were higher than ~92% of the WHODAS 2.0 population norms across the general population of 10 countries (e.g., China, India, Slovakia).

**Table 3:** A comparison of the functional scores among respondents across the six domains of the WHODAS 2.0.

**Variables**	**Medical laboratory technologists**		**Medical laboratory technicians/assistants**		**Total**		**Comparison between groups**
	**Mean ±SD**	**Median**	**Mean ±SD**	**Median**	**Mean ±SD**	**Median**	***p*-Value**
Domain 1	3.06 ± 1.32	3	3.27 ± 1.58	3	3.14 ± 1.42	3	*p* = 0.10
Domain 2	2.94 ± 1.35	2	3.37 ± 1.58	3	3.09 ± 1.45	2	*p* < 0.001^***^
Domain 3	2.29 ± 0.86	2	2.31 ± 0.79	2	2.30 ± 0.83	2	*p* = 0.74
Domain 4	3.54 ± 1.46	3	3.71 ± 1.71	3	3.60 ± 1.55	3	*p* = 0.23
Domain 5	4.27 ± 1.60	4	4.52 ± 1.83	4	4.36 ± 1.69	4	*p* = 0.10
Domain 6	4.28 ± 1.91	4	4.49 ± 2.20	4	4.36 ± 2.01	4	*p* = 0.24
Summary	20.38 ± 6.34	19	21.59 ± 7.22	21	20.80 ± 6.68	20	*p* < 0.05^*^

* *p < 0.05,*

*** *p < 0.001*.

The scores of MLT/A in Domain 2 (Mobility-moving and getting around) were higher than MLT (*t* = −3.4358, *df* = 398.62, *p* < 0.001). The summary scores also presented the same result (*t* = −2.1145, *df* = 410.96, *p* < 0.05).

### Impact of COVID-19

The impact of the COVID-19 pandemic was also assessed in Tables 4, 5. Table 4 presents a cross-tabulation of COPSOQ III scores and COVID-19 among MLTs. While cross-tabulation of COPSOQ III scores and COVID-19 among technicians is found in Table 5. For both groups, the majority of the respondents reported the COPSOQ-III domains as “worse than” since COVID-19.

**Table 4:** Cross-tabulation of COPSOQ-III scores and COVID-19 (medical laboratory technologists).

**COPSOQ - domain**	**COPSOQ score**		**Since COVID-19, my response is _____before**			***P*-value**
Quantitative demands		*N* = 443	Better than *n* = 19 (4.3%)	The same as *n* = 143 (32.3%)	Worse than *n* = 281 (63.4%)	*p* < 0.001
	Low		6	98	94	
	High		13	45	187	
Work pace		*N* = 441	Better than *n* = 15 (3.4%)	The same as *n* = 172 (39.0%)	Worse than *n* = 254 (57.6%)	*p* < 0.001
	Low		8	113	109	
	High		7	59	145	
Emotional demands		*N* = 441	Better than *n* = 4 (0.9%)	The same as *n* = 243 (55.1%)	Worse than *n* = 194 (44.0%)	*p* < 0.001
	Low		1	143	46	
	High		3	100	148	
Influence at work		*N* = 442	Better than *n* = 4 (0.9%)	The same as *n* = 279 (63.1%)	Worse than *n* = 159 (36.0%)	*p* = 0.99
	Low		2	132	76	
	High		2	147	83	
Possibilities for development		*N* = 438	Better than *n* = 21 (4.8%)	The same as *n* = 339 (77.4%)	Worse than *n* = 78 (17.8%)	*p* < 0.01
	Low		6	180	54	
	High		15	159	24	
Meaning of work		*N* = 440	Better than *n* = 65 (14.8%)	The same as *n* = 272 (61.8%)	Worse than *n* = 103 (23.4%)	*p* < 0.001
	Low		17	88	65	
	High		48	184	38	
Predictability		*N* = 439	Better than *n* = 11 (2.5%)	The same as *n* = 213 (48.5%)	Worse than *n* = 215 (49.0%)	*p* < 0.001
	Low		1	78	125	
	High		10	135	90	
Recognition		*N* = 441	Better than *n* = 24 (5.4%)	The same as *n* = 205 (46.5%)	Worse than *n* = 212 (48.1%)	*p* < 0.001
	Low		1	50	130	
	High		23	155	82	
Role clarity		*N* = 440	Better than *n* = 9 (2.0%)	The same as *n* = 299 (68.0%)	Worse than *n* = 132 (30.0%)	*p* < 0.001
	Low		5	77	86	
	High		4	222	46	
Role conflict		*N* = 441	Better than *n* = 5 (1.1%)	The same as *n* = 201 (45.6%)	Worse than *n* = 235 (53.3%)	*p* < 0.001
	Low		5	159	110	
	High		0	42	125	
Quality of leadership		*N* = 440	Better than *n* = 18 (4.1%)	The same as *n* = 235 (53.4%)	Worse than *n* = 187 (42.5%)	*p* < 0.001
	Low		1	57	126	
	High		17	178	61	
Social support from colleagues		*N* = 442	Better than *n* = 22 (5.0%)	The same as *n* = 307 (69.4%)	Worse than *n* = 113 (25.6%)	*p* < 0.001
	Low		2	86	78	
	High		20	221	35	
Social support from supervisor		*N* = 440	Better than *n* = 19 (4.3%)	The same as *n* = 271 (61.6%)	Worse than *n* = 150 (34.1%)	*p* < 0.001
	Low		3	122	119	
	High		16	149	31	
Sense of community at work		*N* = 440	Better than *n* = 21 (4.8%)	The same as *n* = 288 (65.4%)	Worse than *n* = 131 (29.8%)	*p* < 0.001
	Low		0	44	78	
	High		21	244	53	
Insecurity over working Conditions		*N* = 439	Better than *n* = 68 (15.5%)	The same as *n* = 285 (64.9%)	Worse than *n* = 86 (19.6%)	*p* < 0.001
	Low		54	187	18	
	High		14	98	68	
Vertical trust		*N* = 441	Better than *n* = 19 (4.3%)	The same as *n* = 259 (58.7%)	Worse than *n* = 163 (37.0%)	*p* < 0.001
	Low		4	63	97	
	High		15	196	66	
Organizational justice		*N* = 440	Better than *n* = 4 (0.9%)	The same as *n* = 242 (55.0%)	Worse than *n* = 194 (44.1%)	*p* < 0.001
	Low		2	105	153	
	High		2	137	41	
Job satisfaction		*N* = 440	Better than *n* = 12 (2.7%)	The same as *n* = 204 (46.4%)	Worse than *n* = 224 (50.9%)	*p* < 0.001
	Low		1	47	140	
	High		11	157	84	
Work life conflict		*N* = 438	Better than *n* = 4 (0.9%)	The same as *n* = 111 (25.4%)	Worse than *n* = 323 (73.7%)	*p* < 0.001
	Low		1	83	102	
	High		3	28	221	
Self-Rated Health		N = 439	Better than n = 8 (1.8%)	The same as n = 230 (52.4%)	Worse than n = 201 (45.8%)	p < 0.001
	Low		2	89	135	
	High		6	141	66	
Burnout		*N* = 440	Better than *n* = 9 (2.0%)	The same as *n* = 95 (21.6%)	Worse than *n* = 336 (76.4%)	*p* < 0.001
	Low		6	57	98	
	High		3	38	238	
Stress		*N* = 440	Better than *n* = 5 (1.1%)	The same as *n* = 94 (21.4%)	Worse than *n* = 341 (77.5%)	*p* < 0.001
	Low		2	73	160	
	High		3	21	181	
Sexual harassment		*N* = 437	Better than *n* = 2 (0.5%)	The same as *n* = 413 (94.5%)	Worse than *n* = 22 (5.0%)	*p* < 0.001
	Low		2	395	15	
	High		0	18	7	
Threat of violence		*N* = 436	Better than *n* = 2 (0.5%)	The same as *n* = 396 (90.8%)	Worse than *n* = 38 (8.7%)	*p* < 0.001
	Low		1	373	19	
	High		1	23	19	
Physical violence		*N* = 436	Better than *n* = 1 (0.2%)	The same as *n* = 410 (94.1%)	Worse than *n* = 25 (5.7%)	*p* < 0.05
	Low		1	398	21	
	High		0	12	4	

**Table 5:** Cross-tabulation of COPSOQ-III scores and COVID-19 (technicians).

**COPSOQ - domain**	**COPSOQ score**		**Since COVID-19, my response is _____before**			***P-*value**
Quantitative demands		*N* = 245	Better than *n* =20 (8.2%)	The same as *n* = 68 (27.7%)	Worse than *n* =157 (64.1%)	*p* < 0.001
	Low		10	45	59	
	High		10	23	98	
Work pace		*N* = 241	Better than *n* = 16 (6.6%)	The same as *n* = 63 (26.2%)	Worse than *n* =162 (67.2%)	*p* < 0.001
	Low		8	38	54	
	High		8	25	108	
Emotional demands		*N* = 239	Better than *n* = 13 (5.4%)	The same as *n* = 101 (42.3%)	Worse than *n* = 125 (52.3%)	*p* < 0.001
	Low		7	71	40	
	High		6	30	85	
Influence at work		*N* = 237	Better than *n* = 12 (5.1%)	The same as *n* = 130 (54.8%)	Worse than *n* = 95 (40.1%)	*p* < 0.01
	Low		1	74	60	
	High		11	56	35	
Possibilities for development		*N* = 241	Better than *n* = 27 (11.2%)	The same as *n* = 148 (61.4%)	Worse than *n* = 66 (27.4%)	*p* < 0.001
	Low		4	46	35	
	High		23	102	31	
Meaning of work		*N* = 241	Better than *n* =52 (21.6%)	The same as *n* = 121 (50.2%)	Worse than *n* = 68 (28.2%)	*p* < 0.01
	Low		18	47	43	
	High		34	74	25	
Predictability		*N* = 242	Better than *n* = 16 (6.6%)	The same as *n* = 114 (47.1%)	Worse than *n* = 112 (46.3%)	*p* < 0.001
	Low		0	40	69	
	High		16	74	43	
Recognition		*N* = 243	Better than *n* = 30 (12.4%)	The same as *n* =99 (40.7%)	Worse than *n* = 114 (46.9%)	*p* < 0.001
	Low		1	38	75	
	High		29	61	39	
Role clarity		*N* = 242	Better than *n* = 20 (8.3%)	The same as *n* = 123 (50.8%)	Worse than *n* = 99 (40.9%)	*p* < 0.001
	Low		4	29	66	
	High		16	94	33	
Role conflict		*N* = 242	Better than *n* = 20 (8.3%)	The same as *n* = 87 (35.9%)	Worse than *n* = 135 (55.8%)	*p* < 0.001
	Low		10	58	50	
	High		10	29	85	
Quality of leadership		*N* = 238	Better than *n* = 18 (7.6%)	The same as *n* = 106 (44.5%)	Worse than *n* = 114 (47.9%)	*p* < 0.001
	Low		3	26	64	
	High		15	80	50	
Social support from colleagues		*N* = 240	Better than *n* = 26 (10.8%)	The same as *n* = 135 (56.3%)	Worse than *n* = 79 (32.9%)	*p* < 0.001
	Low		5	40	61	
	High		21	95	18	
Social support from supervisor		*N* = 241	Better than *n* = 26 (10.8%)	The same as *n* = 126 (52.3%)	Worse than *n* = 89 (36.9%)	*p* < 0.001
	Low		6	57	73	
	High		20	69	16	
Sense of community at work		*N* = 236	Better than *n* = 16 (6.8%)	The same as *n* = 134 (56.8%)	Worse than *n* = 86 (36.4%)	*p* < 0.001
	Low		1	31	53	
	High		15	103	33	
Insecurity over working conditions		*N* = 240	Better than *n* = 35 (14.6%)	The same as *n* = 129 (53.7%)	Worse than *n* = 76 (31.7%)	*p* < 0.001
	Low		23	80	26	
	High		12	49	50	
Vertical trust		*N* = 238	Better than *n* = 15 (6.3%)	The same as *n* = 145 (60.9%)	Worse than *n* = 78 (32.8%)	*p* < 0.01
	Low		4	42	40	
	High		11	103	38	
Organizational justice		*N* = 239	Better than *n* = 15 (6.3%)	The same as *n* = 119 (49.8%)	Worse than *n* = 105 (43.9%)	*p* < 0.001
	Low		2	33	68	
	High		13	86	37	
Job satisfaction		*N* = 240	Better than *n* = 14 (5.8%)	The same as *n* = 110 (45.8%)	Worse than *n* = 116 (48.4%)	*p* < 0.001
	Low		1	31	79	
	High		13	79	37	
Work life conflict		*N* = 241	Better than *n* = 14 (5.8%)	The same as *n* = 58 (24.1%)	Worse than *n* = 169 (70.1%)	*p* < 0.001
	Low		8	38	56	
	High		6	20	113	
Self-rated health		*N* = 242	Better than *n* = 9 (3.7%)	The same as *n* = 129 (53.3%)	Worse than *n* = 104 (43.0%)	*p* < 0.001
	Low		3	46	77	
	High		6	83	27	
Burnout		*N* = 243	Better than *n* = 12 (4.9%)	The same as *n* = 51 (21.0%)	Worse than *n* = 180 (74.1%)	*p* < 0.001
	Low		8	32	45	
	High		4	19	135	
Stress		*N* = 242	Better than *n* = 10 (4.1%)	The same as *n* = 51 (21.1%)	Worse than *n* = 181 (74.8%)	*p* < 0.001
	Low		7	39	78	
	High		3	12	103	
Sexual harassment		*N* = 240	Better than *n* = 9 (3.7%)	The same as *n* = 208 (86.7%)	Worse than *n* = 23 (9.6%)	*p* = 0.125
	Low		7	181	17	
	High		2	27	6	
Threat of violence		*N* = 240	Better than *n* = 12 (5.0%)	The same as *n* = 178 (74.2%)	Worse than *n* = 50 (20.8%)	*p* < 0.001
	Low		7	135	11	
	High		5	43	39	
Physical violence		*N* = 240	Better than *n* = 12 (5.0%)	The same as *n* = 193 (80.4%)	Worse than *n* = 35 (14.6%)	*p* < 0.01
	Low		10	158	19	
	High		2	35	16	

In addition, respondents with poor psychological status (e.g., the low group for meaning of work; high group for work pace) were more likely to experience greater pressure under the influence of the epidemic. For example, we found that 33.3% (*n* = 84) MLT with high job satisfaction reported that their job satisfaction was worse than before the start of the pandemic, while 74.5% (*n* = 140) MLT with low job satisfaction reported that their job satisfaction was worse than before the start of the pandemic (*P* < 0.001). Furthermore, we found that 67.22% of MLT/A indicated that they felt worse about work pace than before the start of the pandemic, especially those who experienced faster work pace initially.

## Discussion

The main findings of this study found scores of work pace, emotional demands, role conflicts, job insecurity, insecurity over working conditions and negative acts were higher for MLT/A than MLT. The purpose of the study is to examine the current mental status, functioning and disability of medical laboratory professionals in Ontario and the impact of the COVID-19 pandemic on their mental health. This is the first study to examine the psychosocial risk factors of medical laboratory professionals in Ontario, Canada. The health care workers are working at the scenes and are not patient-facing. However, they are the backbone of the healthcare system in providing critical services in general medical laboratory technology, diagnostic typology, and clinical genetics (37). The fact that most of the medical laboratory professions were women, which was larger than other healthcare workers, like doctors, occupational therapists might account for some high scores in COPSOQ because early studies showed that women were more likely to suffer from mental health problems, e.g., depression, anxiety and burnout, considered because job status could also influence mental health problems. For example, Labrague et al. showed that health workers like nurses who worked part-time reported higher fear scores than full-time workers, thus affecting their psychological and emotional wellbeing and work performance (38). The characteristic of part-time work, including temporary and unstable, also increase job insecurity and leaves little room for career development. Therefore, the high proportion of part-time jobs in MLT/A may also be an important reason for this population's prominent psychological health problem.

In addition, negative behaviors like physical violence and sexual harassment in the workplace was also common among laboratory professions, especially among MLT/A group. These kinds of behaviors will affect an individual's work performance or create an intimidating, hostile, or offensive environment (39). Studies focused on nurses have found that greater work demands and less trust and justice were associated with nurses' experiences of violence (40). Manageable workloads and greater understanding of MLT and MLT/A work responsibilities and a more supportive work environment may reduce negative health outcomes acts for medical laboratory professions.

The high sense of job insecurity and insecurity over working conditions was present, especially in the group of MLT/A; heavy demands of workload, including high quantitative and emotional demands and fast work pace were also reflected in medical laboratory professions, especially MLT. This reflected the current problems in the medical laboratory system: inadequate working conditions, high workload, growing competition among healthcare workers and less safety at work. Health workers in such circumstances are experiencing increased pressures at their workplace due to high working demands or do not have enough time to complete the tasks and feel insecure, despite giving maximum effort.

In addition, respondents with poor psychological status (e.g., the low group for meaning of work; high group for work pace) were more likely to experience greater pressure under the influence of the epidemic, which led to further deterioration of their mental health. For example, we found a third MLT with high job satisfaction reported that their job satisfaction was worse than before the start of the pandemic, while almost three quarters of MLT with low job satisfaction reported feeling worse. This vicious circle suggested the importance of early intervention and early treatment for medical laboratory professions.

We found that the mean scores of WHODAS 2.0 in MLT and MLT/A were higher than approximately 92% of the WHODAS 2.0 population norms across the general population of 10 countries, indicating the extent of disability associated with a psychiatric condition among medical laboratory workers was quite serious than average people. When disability limits a person's daily activities, it is clinically significant to consider the impact (i.e., possible mental problems) disability may cause. Moreover, there is evidence that the increase in the WHODAS 2.0 score was associated with increased comorbidities, in particular those related to mental (depression) and physical health (41).

### Strengths and Limitations

Mental Health, functioning and disability were significant health concerns for medical laboratory professionals in Ontario. At the same time, they did not receive the same attention as other healthcare workers, e.g., doctors or nurses. To raise awareness on this occupational group, we worked closely with the Medical Laboratory Professionals' Association of Ontario (MLPAO) to recruit participants across the province to represent the diversity of the workforce across geography and workplace settings (e.g., hospitals, private laboratories, etc.). Therefore, the sample is well representative of this group in Ontario. We used the COPSOQ-III and WHODAS 2.0, two internationally well-known questionnaires to measure the results, giving a better reference value for our results. Our study also covered a comparison in the mental status before and after COVID-19 in this occupational group, which has not been examined in any previous studies.

We also acknowledge other potential limitations. First, this was a cross-sectional study, so that causality could not be established. Second, the study participants (MLT and MLA/T) were emailed a link to the survey using REDCap at one certain time. As a result, this may lead to bias in that the credibility and validity of email patterns were not as high as face-to-face interviews. In addition, there are different versions of COPSOQ-III that may raise questions about the reliability of the results when comparing the results with other studies. Furthermore, the study did not evaluate or examine other conditions of COVID-19, such as issues regarding families and restrictions. As a result, this limitation does not allow us to capture the full impact of Covid-19.

## Conclusion

The study provides preliminary evidence regarding the workplace mental health outcomes of medical laboratory professionals in Ontario, Canada. The implications of the pandemic reflected on mental health and functioning, specifically the limitations on their psychosocial work environments, have led this study to examine these effects. The scores of work pace, emotional demands, role conflicts, job insecurity, insecurity over working conditions and negative acts were higher for MLT/A than MLT. In terms of their functioning, we observed scores that are higher than the 92% of the normed population. There is a need for robust studies that examine a larger sample across time. The findings may also provide information for governments and employers to address strategies to improve the mental health of laboratory medical professionals.

## Data Availability Statement

The raw data supporting the conclusions of this article will be made available by the authors, without undue reservation.

## Ethics Statement

The studies involving human participants were reviewed and approved by University of Toronto Research Ethics Board. The patients/participants provided their written informed consent to participate in this study.

## Author Contributions

All authors listed have made a substantial, direct, and intellectual contribution to the work and approved it for publication.

## Conflict of Interest

The authors declare that the research was conducted in the absence of any commercial or financial relationships that could be construed as a potential conflict of interest.

## Publisher's Note

All claims expressed in this article are solely those of the authors and do not necessarily represent those of their affiliated organizations, or those of the publisher, the editors and the reviewers. Any product that may be evaluated in this article, or claim that may be made by its manufacturer, is not guaranteed or endorsed by the publisher.

## References

[1] Bandali K Zhu L Gamble PAW. Canada's health human resource challenges: what is the fate of our healthcare heroes? Healthc Manage Forum. (2011) 24:179–83. 10.1016/j.hcmf.2011.04.001 22256513

[2] Medical Laboratory Professionals' Association of Ontario. Report: medical laboratory professionals at a breaking point – two-year analysis. (2022). Available online at: https://www.mlpao.org/_files/ugd/691355_870e5fae53f34d0cba723820bac97c13.pdf

[3] Maunder RG Heeney ND Strudwick G Shin HD O'Neill B Young N. Burnout in Hospital-Based Healthcare Workers during COVID-19. Ontario: Science Briefs of the Ontario COVID-19 Science Advisory Table (2021).

[4] Roslan NS Yusoff MSB Asrenee AR Morgan K. Burnout prevalence and its associated factors among Malaysian healthcare workers during COVID-19 pandemic: an embedded mixed-method study. Healthcare. (2021) 9:1–90. 10.3390/healthcare9010090 33477380 PMC7829836

[5] West CP Dyrbye LN Sinsky C Trockel M Tutty M Nedelec L. Resilience and burnout among physicians and the general US working population. JAMA Net Open. (2020). 3:e209385–96. 10.1001/jamanetworkopen.2020.9385 32614425 PMC7333021

[6] Woo T Ho R Tang A Tam W. Global prevalence of burnout symptoms among nurses: a systematic review and meta-analysis. J Psychiatr Res. (2020) 123:9–20. 10.1016/j.jpsychires.2019.12.015 32007680

[7] Medical Laboratory Professionals' Association of Ontario. Medical Laboratory Shortage. Hamilton, ON (2019).

[8] Bainbridge L Davidson K Loranger L. Burnout Among Alberta physiotherapists: A White Paper (2017).

[9] Dyrbye LN Shanafelt TD Johnson PO Johnson LA Satele D West CP. cross-sectional study exploring the relationship between burnout, absenteeism, and job performance among American nurses. BMC Nurs. (2019) 18:1–57. 10.1186/s12912-019-0382-7 31768129 PMC6873742

[10] Wilkins K. Work stress among health care providers. Health Rep. (2007) 18:33–6. 18074995

[11] Rotenstein LS Torre M Ramos MA Rosales RC Guille C Sen S. Prevalence of burnout among physicians. JAMA. (2018) 320:1131–51. 10.1001/jama.2018.12777 30326495 PMC6233645

[12] Sanghera J Pattani N Hashmi Y Varley KF Cheruvu MS Bradley A. The impact of SARS-CoV-2 on the mental health of healthcare workers in a hospital setting—a systematic review. J Occup Health. (2020) 62:1–16. 10.1002/1348-9585.12175 33131192 PMC7603426

[13] Painter J Akroyd D Elliot S Adams RD. Burnout among occupational therapists. Occup Ther Health Care. (2003) 17:63–78. 10.1080/J003v17n01_06 23941190

[14] Poulsen AA Meredith P Khan A Henderson J Castrisos V Khan SR. Burnout and work engagement in occupational therapists. Br J Occup Ther. (2014) 77:156–64. 10.4276/030802214X1394103626662130103359

[15] Scanlan JN Hazelton T. Relationships between job satisfaction, burnout, professional identity and meaningfulness of work activities for occupational therapists working in mental health. Aust Occup Ther J. (2019) 66:581–90. 10.1111/1440-1630.12596 31304598

[16] Sanchez-Gomez M Giorgi G Finstad GL Urbini F Foti G Mucci N. COVID-19 pandemic as a traumatic event and its associations with fear and mental health: a cognitive-activation approach. Int J Environ Res Public Health. (2021) 18:1–14. 10.3390/ijerph18147422 34299873 PMC8303753

[17] Torales J O'Higgins M Castaldelli-Maia JM Ventriglio A. The outbreak of COVID-19 coronavirus and its impact on global mental health. Int J Soc Psychiatry. (2020) 66:317–20. 10.1177/0020764020915212 32233719

[18] Smallwood N Karimi L Bismark M Putland M Johnson D Dharmage SC. High levels of psychosocial distress among Australian frontline healthcare workers during the COVID-19 pandemic: a cross-sectional survey. Gen Psychiatry. (2021) 34:1–11. 10.1136/gpsych-2021-100577 34514332 PMC8423519

[19] Denning M Goh ET Tan B Kanneganti A Almonte M Scott A. Determinants of burnout and other aspects of psychological well-being in healthcare workers during the Covid-19 pandemic: A multinational cross-sectional study. PLoS ONE. (2021) 16:1–18. 10.1371/journal.pone.0238666 33861739 PMC8051812

[20] Barello S Palamenghi L Graffigna G. Burnout and somatic symptoms among frontline healthcare professionals at the peak of the Italian COVID-19 pandemic. Psychiatry Res. (2020) 290:113129–33. 10.1016/j.psychres.2020.113129 32485487 PMC7255285

[21] Chor WPD Ng WM Cheng L Situ W Chong JW Ng LYA. Burnout amongst emergency healthcare workers during the COVID-19 pandemic: a multi-center study. Am J Emerg Med. (2021) 46:700–2. 10.1016/j.ajem.2020.10.040 33129643 PMC7584488

[22] Estes BA Varghese JJ Jacques J Naidu SS. Logistical, financial, and psychological impact of the COVID-19 pandemic on cardiac catheterization lab nurses and technologists: A US National Survey. J Invasive Cardiol. (2021) 33:E9–15. 10.5709/ce.1897-9254.433 33279880

[23] Pazmiño Erazo EE Alvear Velásquez MJ Saltos Chávez IG Pazmiño Pullas DE. Factors associated with psychiatric adverse effects in healthcare personnel during the COVID-19 pandemic in Ecuador. Revista Colombiana de Psiquiatría. (2021) 50:166–75. 10.1016/j.rcpeng.2020.12.001 34481796 PMC8352659

[24] Medical Laboratory Professionals' Association of Ontario. About The MLPAO (2021). Available online at: https://www.mlpao.org/about

[25] Booker LA Sletten TL Alvaro PK Barnes M Collins A Chai-Coetzer CL. Exploring the associations between shift work disorder, depression, anxiety and sick leave taken amongst nurses. J Sleep Res. (2020) 29:1–9. 10.1111/jsr.12872 31144389

[26] Cocker F Joss N. Compassion fatigue among healthcare, emergency and community service workers: a systematic review. Int J Environ Res Public Health. (2016) 13:1–18. 10.3390/ijerph13060618 27338436 PMC4924075

[27] Duarte J Pinto-Gouveia J. The role of psychological factors in oncology nurses' burnout and compassion fatigue symptoms. Eur J Oncol Nurs. (2017) 28:114–21. 10.1016/j.ejon.2017.04.002 28478848

[28] Hall LH Johnson J Watt I Tsipa A O'Connor DB. Healthcare staff wellbeing, burnout, and patient safety: a systematic review. PLoS ONE. (2016) 11:1–12. 10.1371/journal.pone.0159015 27391946 PMC4938539

[29] Harris PA Taylor R Minor BL Elliott V Fernandez M O'Neal L. The REDCap consortium: building an international community of software platform partners. J Biomed Inform. (2019) 95:1–24. 10.1016/j.jbi.2019.103208 31078660 PMC7254481

[30] Taherdoost H. Determining sample size; how to calculate survey sample size. Int J Econ Manag Syst. (2017) 2:237–9.

[31] Burr H Berthelsen H Moncada S Nübling M Dupret E Demiral Y. The third version of the copenhagen psychosocial questionnaire. Saf Health Work. (2019) 10:482–503. 10.1016/j.shaw.2019.10.002 31890332 PMC6933167

[32] Ustun TB Kostanjsek N Chatterji S Rehm J. Measuring Health and Disability: Manual for WHO Disability Assessment Schedule (WHODAS 2.0). Geneva: World Health Organization (2010). p. 1–88. 10.2471/BLT.09.067231 PMC2971503 21076562

[33] MacLeod MA Tremblay PF Graham K Bernards S Rehm J Wells S. Psychometric properties and a latent class analysis of the 12-item world health organization disability assessment schedule 20 (WHODAS 20) in a pooled dataset of community samples International. Int J Methods Psychiatr Res. (2016) 25:243–54. 10.1002/mpr.1523 27634553 PMC6860311

[34] Pösl M Cieza A Stucki G. Psychometric properties of the WHODASII in rehabilitation patients. Qual Life Res. (2007) 16:1521–31. 10.1007/s11136-007-9259-4 17828578

[35] Chan BKC. Data Analysis Using R Programming. In: Biostatistics for Human Genetic Epidemiology. Sunnyvale, CA: Springer International Publishing (2018). p. 47–122.

[36] Slany C Schütte S Chastang JF Parent-Thirion A Vermeylen G Niedhammer I. Psychosocial work factors and long sickness absence in Europe. Int J Occup Environ Health. (2014) 20:16–25. 10.1179/2049396713Y.0000000048 24176393 PMC4137803

[37] Canadian Institute for Health Information. Medical Laboratory Technologists. Canadian Institute for Health Information (2021).

[38] Labrague LJ Santos JAA. Fear of COVID-19, psychological distress, work satisfaction and turnover intention among frontline nurses. J Nurs Manag. (2021) 29:395–403. 10.1111/jonm.13168 32985046 PMC7537256

[39] Nukala M Freedman-Weiss M Yoo P Smeds MR. Sexual harassment in vascular surgery training programs. Ann Vasc Surg. (2020) 62:92–7. 10.1016/j.avsg.2019.05.011 31220589

[40] Park M Cho SH Hong HJ. Prevalence and perpetrators of workplace violence by nursing unit and the relationship between violence and the perceived work environment. J Nurs Scholarsh. (2015) 47:87–95. 10.1111/jnu.12112 25352254

[41] Ferrer MLP Perracini MR Rebustini F Buchalla CM. WHODAS 2.0-BO. Revista de Saúde Pública. (2019) 53:19. 10.11606/S1518-8787.2019053000586 30726500 PMC6394378

